# A novel *MYH14* mutation in a Chinese family with autosomal dominant nonsyndromic hearing loss

**DOI:** 10.1186/s12881-020-01086-y

**Published:** 2020-07-25

**Authors:** Mingming Wang, Yicui Zhou, Fengguo Zhang, Zhaomin Fan, Xiaohui Bai, Haibo Wang

**Affiliations:** 1 Department of Otorhinolaryngology Head and Neck Surgery, Shandong Provincial ENT Hospital, Cheeloo College of Medicine, Shandong University, Jinan, China; 2 Department of Otorhinolaryngology Head and Neck Surgery, the First Affiliated Hospital of USTC, Division of Life Sciences and Medicine, University of Science and Technology of China, Hefei, China; 3 Shandong Institute of Otolaryngology, Shandong Provincial ENT Hospital, Cheeloo College of Medicine, Shandong University, Jinan, China; 4 Department of Clinical Laboratory, Shandong Provincial Hospital, Cheeloo College of Medicine, Shandong University, Jinan, China

**Keywords:** MYH14, Mutation, Hearing loss, Targeted next-generation sequencing, Family

## Abstract

**Background:**

*MYH14* gene mutations have been suggested to be associated with nonsyndromic/syndromic sensorineural hearing loss. It has been reported that mutations in *MYH14* can result in autosomal dominant nonsyndromic deafness-4A (DFNA4).

**Methods:**

In this study, we examined a four-generation Han Chinese family with nonsyndromic hearing loss. Targeted next-generation sequencing of deafness genes was employed to identify the pathogenic variant. Sanger sequencing and PCR-RFLP analysis were performed in affected members of this family and 200 normal controls to further confirm the mutation.

**Results:**

Four members of this family were diagnosed as nonsyndromic bilateral sensorineural hearing loss with postlingual onset and progressive impairment. A novel missense variant, c.5417C > A (p.A1806D), in *MYH14* in the tail domain of NMH II C was successfully identified as the pathogenic cause in three affected individuals. The family member II-5 was suggested to have noise-induced deafness.

**Conclusion:**

In this study, a novel missense mutation, c.5417C > A (p.A1806D), in *MYH14* that led to postlingual nonsyndromic autosomal dominant SNHL were identified. The findings broadened the phenotype spectrum of *MYH14* and highlighted the combined application of gene capture and Sanger sequencing is an efficient approach to screen pathogenic variants associated with genetic diseases.

## Background

The *MYH14* gene, also known as nonmuscle heavy chain II C (NMHCII-C), encodes one of the myosin members. In motility processes, such as cytoskeleton rearrangement, organelle translocation and ion channel gating, *MYH14* is indicated to play a major role in the directed movement of cell components along actin filaments by ATP hydrolysis to generate force [1–3]. *MYH14* (NMHCII-C) has been characterized recently, but there is limited information on its biological functions. The *MYH14* gene is located on the chromosome 19q13.33, contains 41 exons and encodes a protein with 1995 amino acids, which contains a myosin head region, two IQ domains, an N-terminal myosin domain, and a C-terminal myosin tail [4]. A polar structure is formed by the dimerization of heavy chains. The N-terminal domain has two globular heads that are ATP-binding and actin-binding regions and are essential for motility, while the helical C-terminus is a singular rod-like tail that can polymerize molecules into bipolar filaments in nonmuscle and muscle cells [5]. The *MYH14* gene was reported to be related to peripheral neuropathy, myopathy, hoarseness and hearing loss (PNMHH) [6], and the *MYH14* protein is expressed widely within cochlear tissues, such as the organ of Corti, spiral prominence epithelium, stria vascularis, and cochlear duct. Low expression was detectable in the Reissner’s membrane and spiral ligament, but no expression was detected in vestibular epithelia in mice [7]. *MYH14* plays a part in neurogenesis and maintenance of apical cell junctions in epithelial cells within the cochlea [8, 9]. The expression of *MYH14* in mice and humans is generally higher in adults than in adolescents [4, 10, 11].

Hereditary sensorineural hearing loss (SNHL) can be classified into nonsyndromic and syndromic hearing loss. 75% of hereditary SNHL cases are nonsyndromic. Eighty percent of nonsyndromic SNHL is in an autosomal recessive (AR) inheritance pattern and tends to result in severe SNHL with prelingual onset, whereas autosomal dominant (AD) inherited deafness usually leads to mild and flat downsloping hearing loss with postlingual onset [12, 13]. Pathogenic variants of the *MYH14* gene can cause either syndromic or nonsyndromic SNHL and can be identified as the pathogenic factor DFNA4A [7, 14–16]. To date, missense and nonsense variants of the *MYH14* gene have been reported in families with mild to severe degrees of AD SNHL, which usually manifests with milder hearing loss and later onset than AR inherited hearing loss. A recent study showed the *MYH14* gene may be an essential gene related to nonsyndromic AD SNHL [17]. However, the physiological link between *MYH14* mutations and sensorineural hearing loss is still unclear.

In the current study, we investigated pathogenic variants in a four-generation Han Chinese family suffering from nonsyndromic sensorineural hearing loss with postlingual onset. We screened 127 genes known to be related to deafness by gene capture and next-generation sequencing and identified the novel variant c.5417C > A (p.A1806D) in *MYH14* on the tail domain of NMH II C. By reporting novel pathogenic variants, the phenotypic spectrum of the *MYH14* gene could be broadened in the field of hereditary hearing loss.

## Methods

### Subjects

Fifteen members from a four-generation Han Chinese family were enrolled in this study, and four of them were diagnosed as sensorineural hearing loss with postlingual onset and progressive impairment. All participants agreed to undergo clinical examinations as well as audiometric and vestibular function evaluations, including otoscopy, tympanometry, pure-tone audiometry (PTA), speech recognition score (above the hearing threshold of 30 dB HL) evaluation, auditory brainstem response (ABR) evaluation, distortion product otoacoustic emission (DPOAE) recording, a vestibular bithermal caloric test, and ocular/cervical vestibular evoked myogenic potential (o/c VEMP) evaluation. High-resolution computed tomography (HRCT) to image the temporal bone and brain MRI were used to define cochleovestibular malformation. The hearing loss onset of members IV-1 and IV-2 at the ages of 3 and 1 was evaluated by behavior observation audiometry. The degree of SNHL was classified into five grades, namely, normal hearing (PTA ≤ 25 dB HL) and mild (26 ~ 40 dB HL), moderate (41 ~ 60 dB HL), severe (61 ~ 80 dB HL), and profound hearing loss (PTA ≥81 dB HL), based on the average PTA threshold applied at 250, 500, 1000, 2000, 4000, and 8000 Hz. The study protocol was permitted by the Ethics Committee of Shandong Provincial ENT Hospital (XYK20140101), and adhered to the Declaration of Helsinki principles. Informed written consent was obtained from each subject or, in the case of minors, from their parents.

### Targeted gene capture and next-generation sequencing

To investigate the pathogenic mutations in this family, a genomic DNA (gDNA) purification kit (Axygen, San Francisco, CA) was applied to extract gDNA from peripheral blood by the manufacturer’s instructions. Targeted deafness gene capture and next-generation sequencing were performed in the probands to screen 127 genes [18] (Table S1 in Supplementary Material) related to nonsyndromic and syndromic hearing loss by BGI (Beijing Genomics Institute, Shenzhen, China) through a standardized next-generation capture sequencing platform [19]. All exons and the surrounding ±10 bp in the flanking intronic regions of the 127 deafness genes were sequenced. An E210 DNA-shearing devise (Covaris S2, Massachusetts, USA) was employed to fragment the whole gDNA. The library fragment sizes were mainly distributed within 250 ~ 300 bp. Adapter ligation, end repair and adenylation were performed for library preparation in accordance with the standard Illumina protocols. An Illumina HiSeq2000 analyzer was used to capture targeted DNA fragments [20], and image analysis, base calling, and error estimation were carried out to generate primary data by the Illumina Pipeline (version 1.3.4). Using BWA MultiVision software, reads were aligned to the NCBI37/hg19 assembly. SOAPsnp software was used as a reference for recorded SNPs, indels and splice variants. The other variant types, including CNVs and complicated genomic rearrangements, were not included. Mutations were detected by the GATK Indel Genotyper. Databases including NCBI dbSNP, the 1000 Genomes database, the HapMap database and BGI’s own databases were used as references [21]. The pathogenic variations were scanned using the 1000 Genomes database (Phase I) (http://www.1000genomes.org) and the HapMap database (combined Phases II and III). The American Medical Genetics and Genomics Guide were referred to interpret and filtered the data based on a simple AD inheritance pattern by keeping only the heterozygous variants. Remaining variants were then filtered using a low likelihood of adverse functionality on the basis of the mutation type, as assessed with pathogenicity prediction tools, including Mutation Taster, SIFT and PolyPhen 2. The American College of Medical Genetics and Genomics (ACMG) guidelines were used to define the DNA variants.

### Mutation verification

PCR amplification and Sanger sequencing were carried out to verify candidate variants. As previously described [22], PCR was executed in a 50 μl reaction mixture. The forward and reverse primers were 5′- GATGGTCTCGTGGACTTAT-3′ and 5′-TGTGGAGGTCACCTTTCT-3′ for the *MYH14* c.5417C > A mutation and 5′- TTGGTGTTTGCTCAGGAAGA-3′ and 5′-GGCCTACAGGGGTTTCAAAT-3′ for the *GJB2* c.109G > A mutation. PCR products were purified and sequenced by the ABI 3730XL Genetic Analyzer. Analysis of sequencing data was performed by DNASTAR sequence analysis software. PCR-RFLP was employed to confirm the c.5417C > A (p.A1806D) mutation in the *MYH14* gene. Primers flanking the candidate mutation were designed to amplify a 512-bp PCR product, which was then digested into 308-bp and 204-bp fragments by the restriction enzyme Eco130I (Thermo Scientific, USA). The digested products were analyzed through electrophoresis by using a 2% agarose gel. *MYH14* mRNA (RefSeq NM_001077186.2) was used to align the sequences as a reference by Lasergene SeqMan software. Multiple-sequence alignment was conducted to perform phylogenetic analysis by ClustalW2 software. The Multiple sequences aligned included NP_001070654.1 (*Homo sapiens*), NP_001094160.1 (*Rattus norvegicus*), NP_001258467.1 (*Mus musculus*), XP_014980128.1 (*Macaca mulatta*), XP_003316592.1 (*Pan troglodytes*), XP_023988172.1 (*Physeter catodon*), and XP_010813541.1 (*Bos taurus*).

## Results

### Clinical findings

**Fig. 1:**
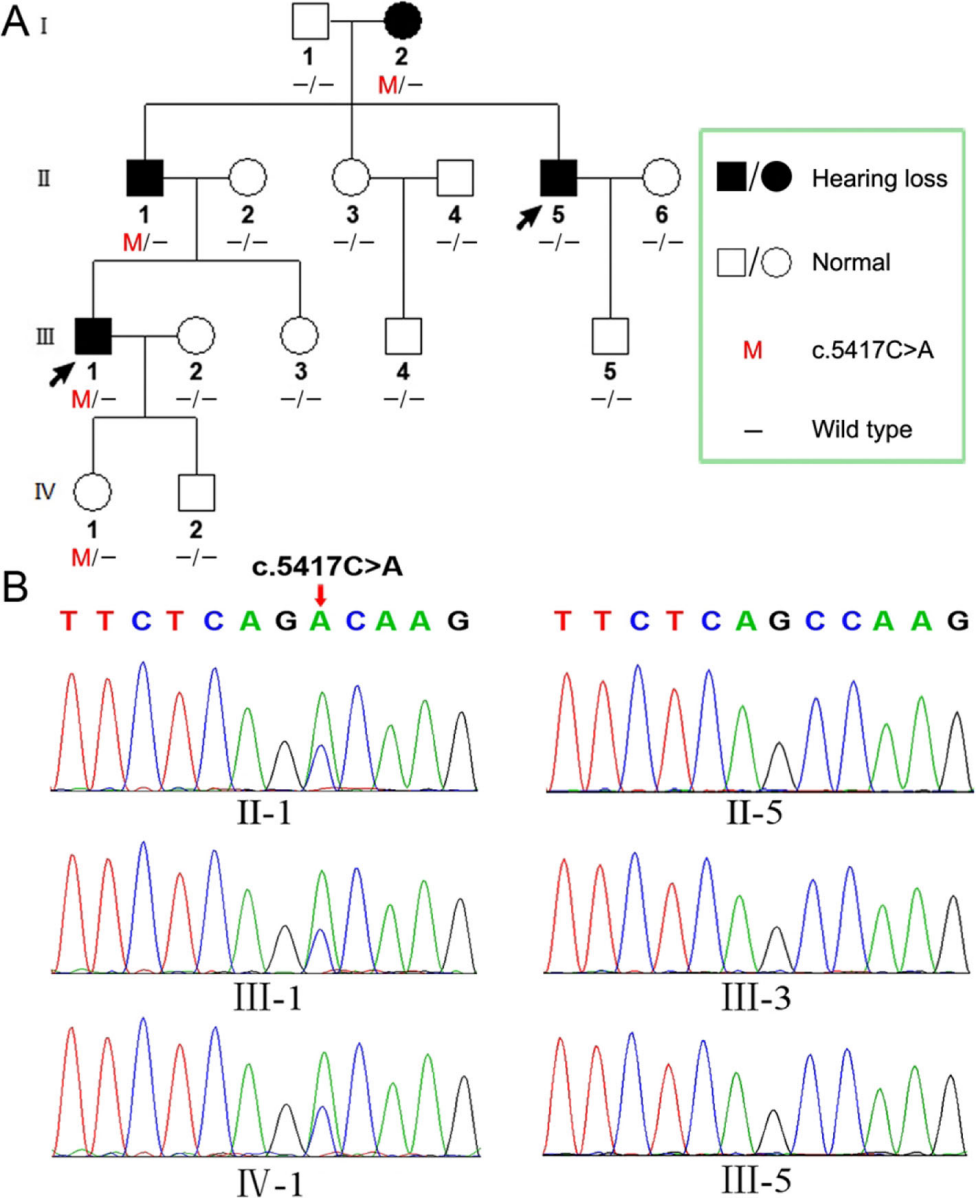
**a** The genealogical tree of the Chinese pedigree. The probands are indicated by arrows. **b** the DNA sequencing profile shows the c.5417C > A mutation in the *MYH14* in II-1, III-1 and IV-1

**Fig. 2:**
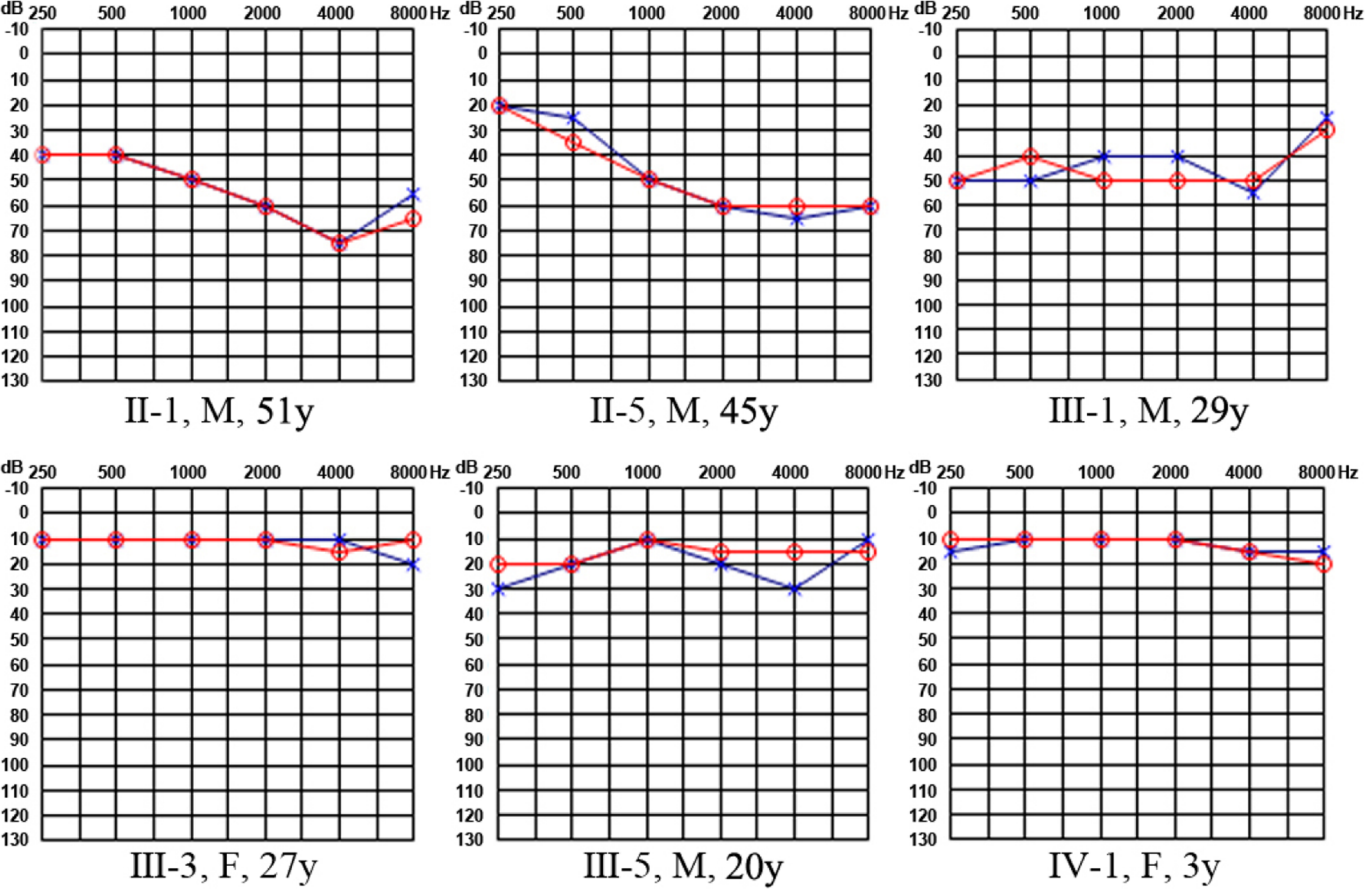
Bilateral pure tone audiograms from individuals in this family. Individual IV-1 was examined by behavior observation audiometry (circles in the audiograms represent the right air conduction thresholds, and crosses, the left ear)

A four-generation Chinese family with 15 members from Shandong Province, China, was enrolled in the study. The family members of II-5 and III-1 were probands, and the pedigree is shown in Fig. 1 according to the statements of the participants. Four members of this Han Chinese family suffered from similar symptoms of tinnitus and hearing loss and presented bilateral and symmetric hearing impairment with postlingual onset and progressive impairment. The age of onset widely ranged from teens to thirties. The PTA results for the members with symptoms showed that moderate sensorineural hearing loss affected almost all frequencies (Fig. 2).

One of the probands (patient III-1) in this family was a 30-year-old male suffering from bilateral nonsyndromic hearing impairment that started in his teens and developed progressively. The PTA performed in our hospital at the age of 29 years showed moderate and flat downsloping SNHL in both ears. The maximum speech recognition scores (SRSs) were 36% in the left and 32% in the right ear. The vestibular function tests showed coordination, and III-1 never complained of vertigo or dizziness. Another proband (II-5) had bilateral nonsyndromic hearing loss with onset in his thirties. However, he has worked in a glassware factory since his twenties and was exposed to noise more than 8 h every day over 10 years.

Constant binaural tinnitus was a common symptom among these patients. The tinnitus volume was at 5 ~ 10 dB HL, which was higher than the auditory threshold of 3 ~ 6 kHz. Family member II-1 had bilateral nonsyndromic hearing loss with onset in his thirties, and member I-2 was affected by progressive hearing impairment since her twenties. The two affected members failed to pass the DPOAE test bilaterally at most or all frequencies, and their ABR results were consistent with the PTA results, which revealed moderate sensorineural hearing loss (Fig. 2). Vestibular tests showed no obvious dysfunction in II-1 and II-5. The clinical examinations are listed in Table 1. The unaffected participants’ examinations and evaluations were normal, and no clinical syndromes were identified.

**Table 1:** Clinical features of individuals with sensorineural hearing loss in this family

Examinations	II-1	II-5	III-1
PTA threshold (dB HL)	56.25 / 58.75 (left/right)	50.00 / 51.25 (left/right)	56.25 / 58.75 (left/right)
SRS (%)	64 / 64 (left/right)	92 / 88 (left/right)	36 / 32 (left/right)
Tympanometry	“A” type	“A” type	“A” type
ABR threshold (dB nHL)	70 (both)	70/60 (left/right)	60 (both)
DPOAE	Absent (both)	Absent (both)	Absent (both)
Cochlear microphone	No elicited	No elicited	No elicited
Vestibular bithermal calorid test	Normal	Normal	Labyrinth reactivity lower (8.4°/s)
cVEMP	Normal	Normal	No wave
oVEMP	Low-amplitude in both ears	Low-amplitude in left and normal in right	N1 and P1 waves recorded only in right ear
Optic nerve electroretinogram	Normal	Normal	Normal
Temporal bone HRCT	Normal	Normal	Normal
Brain MRI	Normal	Normal	Normal

### Targeted gene capture and next-generation sequencing

To identify the pathogenic variants in this Chinese family, targeted gene capture sequencing was performed in the two probands (II-5 and III-1). All variants were filtered through NCBI dbSNP, the HapMap database, the 1000 Genomes database and the in-house databases. The c.5417C > A mutation in *MYH14* and c.109G > A mutation in *GJB2* were detected. Next, Sanger sequencing was used to screen these two candidate variants in 15 family members (Supplementary Table 2). Based on the inheritance pattern and characteristics of deafness, combined with the results of Sanger sequencing, one heterozygous missense mutation c.5417C > A (p.A1806D) was identified, which cosegregated with hearing impairment in this family and was inherited in an autosomal dominant pattern. The c.5417C > A (p.A1806D) variant, located in the tail domain of the *MYH14* gene, causes a change from alanine to aspartic acid at codon 1806. The deleterious and pathogenic aspects of the mutation c.5417C > A (p.A1806D) are listed in Table 2.

**Table 2:** Characteristics of the *MYH14* variant, analysis of predicted protein structure and disease-causing effects

		Variation										
Gene	Exon	Nucleotide^a^	Amino acid^a^	Type	Status	SIFT	PolyPhen 2	Mutation Taster	1000G	DVD	Clinvar	LOVD3
MYH14	39	c.5417C > A	p.A1806D	missense	Heter	Damaging	Damaging	Disease causing	–	–	–	–

*c* variation at cDNA level, *Clinvar* Clinvar Database, *DVD* Deafness Variation Database, *1000G* 1000 Genomes, *Heter* heterozygote, *LOVD3* Leiden Open Variation Database, *p* variation at protein level, *MYH14* myosin heavy chain 14 (NM_001077186), *PolyPhen 2* Polymorphism Phenotyping v2, *SIFT* sorts intolerant from tolerant

^a^All nucleotide and amino acid are abbreviated according to the International Union of Pure and Applied Chemistry

### A novel missense mutation identified in MYH14

**Fig. 3:**
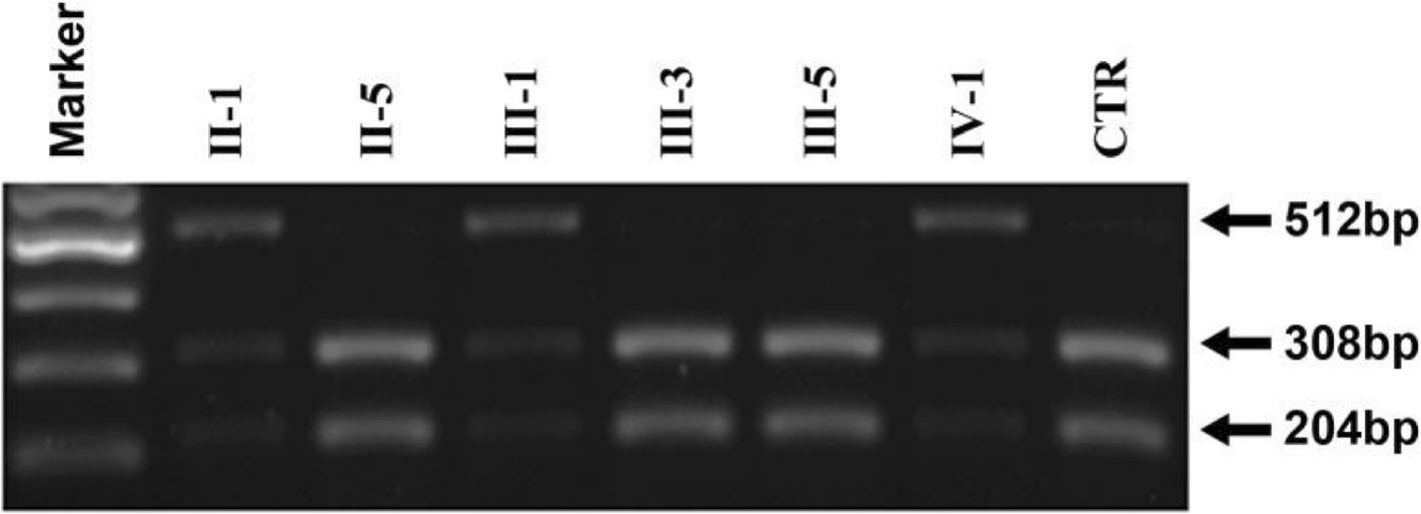
PCR-RFLP results confirms the identification of c.5417C > A mutation in the *MYH14* gene. 512-bp PCR products around c.5417C region were digested with *Eco130*I and analyzed by electrophoresis through a 2% agarose gel stained with ethidium bromide

**Fig. 4:**
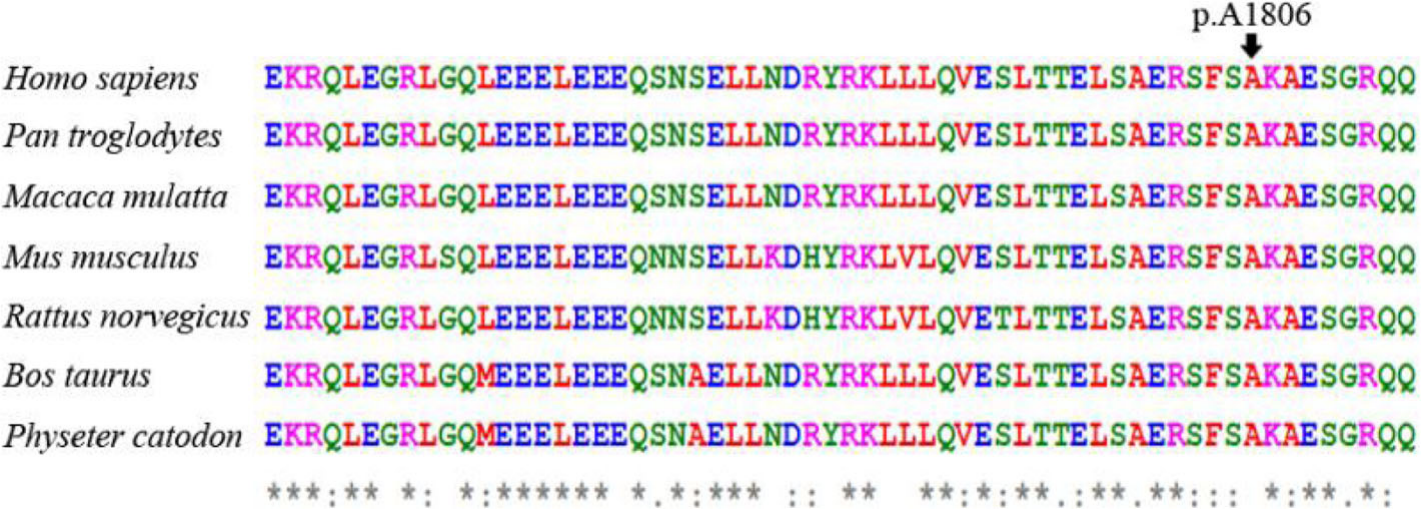
The protein alignment shows conservation across seven species. The mutation c.5417C > A occurred at evolutionarily conserved amino acids

Sanger sequencing was employed to verify this candidate mutation in all the family members. We found that three affected adults (I-2, II-1, III-1) and the three-year-old daughter (IV-1) of patient III-1 harbored this heterozygous variant c.5417C > A (p.A1806D). None of the other subjects carried this variant (as shown in Fig. 1). Considering the age of onset of hearing loss in this family, the three-year-old daughter of patient III-1 could have normal hearing despite carrying the pathogenic variant. Additionally, consistent with our previous diagnosis, the hearing impairment of patient II-5 was caused by noise exposure, not by genetic mutation. To test the frequency of the c.5417C > A (p.A1806D) variant in the general population, Sanger sequencing was performed in 200 people with normal hearing, and no mutation was found at this site. Subsequently, we further analyzed the presence of the c.5417C > A (p.A1806D) mutation by RFLP. As shown in Fig. 3, the PCR products from normal members were digested into two fragments (308 bp and 204 bp), while the PCR products from the affected members (II-1, III-1) and the daughter (IV-1) of III-1 were digested into three fragments (308 bp, 204 bp and 512 bp) because of the presence of the c.5417C > A (p.A1806D) mutation. Figure 4 lists *MYH14* alignments from different species, including *Homo sapiens*, *Rattus norvegicus*, *Mus musculus*, *Macaca mulatta*, *Pan troglodytes*, *Physeter catodon* and *Bos taurus.* The high conservation of alanine at position 1806 in *MYH14* demonstrates that it might play a key role in the biological function, and a variant at this amino acid position could be pathogenic.

## Discussion

*MYH14* is involved in many motility processes, such as organelle translocation, cytoskeleton rearrangement and ion channel gating. The protein is more abundant in adult tissues, with an approximately six-fold increase between E12.5 and E16.5 in the mouse cochlear duct [11, 23]. It has an active role in cellular remodeling in the epithelium of cochlear sensory cells. Inhibition of the *MYH14* gene can lead to defects in extension of the cochlear duct, which may be the physiological mechanism of hearing impairment in subjects who carry *MYH14* gene mutations [24]. Mutations in the *MYH14* gene can result in peripheral neuropathy, hoarseness, myopathy, and hearing loss [6].

In our study, we examined a four-generation Han Chinese family presenting with autosomal dominant nonsyndromic deafness and identified a novel missense mutation, c.5417C > A (p.A1806D), in the *MYH14* gene by using targeted gene capture and Sanger sequencing. Patient III-1 and his father (II-1) and grandmother (I-2) suffered from bilaterally symmetric SNHL with postlingual onset at ages ranging from teens to thirties, and their hearing loss gradually accelerated with aging. All three affected members were identified as carrying this novel variant c.5417C > A (p.A1806D) in the *MYH14* gene, but this variant was absent in 200 normal controls and all the other unaffected family members except for patient III-1’s three-year-old daughter (IV-1). Considering the late onset of SNHL in her family, we will closely follow-up with the three-year-old girl. Interestingly, the SRS of III-1 was much worse than that of II-1. This finding might be related to the age of onset of hearing loss, which exhibited a slow progression. We should follow-up with these individuals from the family.

Patient II-5 worked in a glassware factory and was exposed to noise for more than 8 h every day over 10 years since his twenties. His PTA hearing test showed an increase in medium-high frequencies but normal levels in low frequencies bilaterally, and the SRS values were 92% for the left side and 88% for the right side, which also suggested noise-induced hearing loss. The syndromic features associated with the brain, heart and kidney in this family were excluded.

Although there were no clinical symptoms of vestibular dysfunction in any of the members of this Chinese family, the functional evaluation in proband III-1 showed vestibular impairment. Previous studies reported that there was no detectable expression of *MYH14* in vestibular epithelia in mice [7]. Therefore, despite the presence of a missense mutation in *MYH14*, patient III-1 did not show any clinical vestibular symptoms, which could be due to functional compensation of the vestibular system during the long course of disease.

Table 3 shows all the previously reported pathogenic or likely pathogenic variants in the *MYH14* gene that cause mild to severe degrees of nonsyndromic and progressive SNHL with postlingual onset, as well as the novel variant identified in the current study. Interestingly, among these missense mutations, only one mutation has been reported to lead to syndromic hearing loss, but the others cause nonsyndromic hearing loss [26]. In this study, we demonstrated a new missense mutation in *MYH14* that could have led to moderate nonsyndromic SNHL with postlingual onset in a Han Chinese family. There are some East Asian alleles reported in gnomAD, indicating that this mutation may be an allele in Asians that causes hearing loss. The evolutionary conservation of the alanine residue at codon 1806 (A1806D) in *MYH14* indicates to us that the variant could be pathogenic and cosegregated with the disease in this family. PCR-RFLP was performed to further confirm the results in all the family members and 200 individuals with normal hearing. However, the mechanism by which the c.5417C > A (p.A1806D) variant is associated with hearing loss is not clear. Further functional researches are required to analyze the pathophysiologic mechanism underlying the auditory defects caused by the variant in *MYH14* by constructing a transgenic mouse model.

**Table 3:** *MYH14* gene mutations related with sensorineural hearing loss

Exon	Mutation^**a**^	Amino acid change	Type of variant	Reference	DVD	ClinVar	LOVD3
E-1	c.20 C > A	S7X	Nonsense	Donaudy(2004) [7]	**+**	**+**	**+**
E-2	c.73 C > T	G25X	Nonsense	Kim(2016) [17]	–	–	–
E-2	c.359 C > T	S120L	Missense	Yang(2010) [16]	**+**	**+**	**+**
E-2	c.466C > T	S120L	Missense	Yang(2005) [3]	–	–	–
E-3	c.541 G > A	A181T	Missense	Qing (2014) [25]	**+**	–	**+**
E-3	c.572 A > G	A191G	Missense	Kim(2016) [17]	–	–	–
E-9	c.1126G > T	G376C	Missense	Donaudy(2004) [7]	–	–	–
E-12	c.1314del	P438L	Missense	–	–	–	**+**
E-16	c.2176 C > A	R726S	Missense	Donaudy(2004) [7]	–	–	**+**
E-19	c.2299C > A	A767S	Missense	–	**+**	**+**	–
E-20	c.2569A > C	L857G	Missense	–	–	–	**+**
E-22	c.2594C > T	T865 M	Missense	Qing (2014) [25]	–	–	**+**
E-22	c.2692A > C	L898G	Missense	–	**+**	–	–
E-23	c.2717C > T	T906M	Missense	–	**+**	–	–
E-23	c.2798G > T	A933L	Missense	–	–	–	**+**
E-23	c.2822G > T	R941L	Missense	Choi(2011) [6, 26]	–	–	–
E-24	c.2921G > T	A974L	Missense	–	**+**	**+**	–
E-24	c.2926C > T	L976F	Missense	Donaudy(2004) [7]	–	–	**+**
E-24	c.2971G > A	G991L	Missense	–	–	**+**	–
E-25	c.3049C > T	L1017P	Missense	–	**+**	**+**	–
E-33	c.4780G > A	G1594L	Missense	–	–	–	**+**
E-34	c.4885C > T	A1629C	Missense	–	–	–	**+**
E-35	c.4903G > A	G1635L	Missense	–	**+**	–	–
E-36	c.5008C > T	A1670C	Missense	Vona (2014) [27]	**+**	–	–
E-39	c.5417C > A	A1806D	Missense	**this study**	**-**	**-**	**-**
E-41	c.5893G > T	G1965X	Nonsense	-	-	-	**+**
E-43	c.6016G > T	G2006T	Missense	–	**+**	–	–

*c* variation at cDNA level, *Clinvar* Clinvar Database, *DVD* Deafness Variation Database, *LOVD3* Leiden Open Variation Databas, *MYH14* myosin heavy chain 14 (NM_001077186)

^a^All nucleotide and amino acid are abbreviated according to the International Union of Pure and Applied Chemistry

## Conclusion

This study identified the novel and potentially pathogenic heterozygous missense variant c.5417C > A (p.A1806D) in the *MYH14* gene; this variant is responsible for postlingual nonsyndromic SNHL in a four-generation Han Chinese family. The findings also highlighted the combined application of gene capture and Sanger sequencing is an efficient approach to screen pathogenic variants associated with genetic diseases such as autosomal dominant nonsyndromic deafness.

## Supplementary information

**Additional file 1: Table 1.** Summary of the 127 targeted deafness genes.

**Additional file 2: Table 2.** Candidate variants were verified in the family using Sanger sequencing.

## Data Availability

The raw datasets generated and analysed during the current study are under restricted access from the Key Technology Research and Development Program of Shandong. However, they are available from the corresponding author on reasonable request. The datasets generated and/or analysed during the current study are available in NCBI dbSNP (https://www.ncbi.nlm.nih.gov/snp/?term=), 1000 Genomes (http://www.1000genomes.org), HapMap database (https://www.sanger.ac.uk/resources/downloads/human/hapmap3.html), BGI’s own databases (BGI Inc., Shenzhen, China), Clinvar Database (https://www.ncbi.nlm.nih.gov/), Deafness Variation Database (http://deafnessvariationdatabase.org/gene/MYH14), and Leiden Open Variation Database (https://databases.lovd.nl/shared/genes/MYH14). The accession numbers corresponding to some of the datasets used in this study and obtained from NCBI (National Center for Biotechnology Information) include: NM_001077186.2, NP_001070654.1, NP_001094160.1, NP_001258467.1, XP_014980128.1, XP_003316592.1, XP_023988172.1, XP_010813541.1 and the NCBI37/hg19 assembly (https://www.ncbi.nlm.nih.gov/assembly/GCF_000001405.13/).
