# Supplementary material for: A novel MYH14 mutation in a Chinese family with autosomal dominant nonsyndromic hearing loss
Source: BMC Med Genet. 2020 Jul 25;21:154. doi: 10.1186/s12881-020-01086-y (PMC7382048; doi:10.1186/s12881-020-01086-y)
Supplement: Supplementary file 1 — Additional file 1: Table 1. Summary of the 127 targeted deafness genes. [file 12881_2020_1086_MOESM1_ESM.docx]

**Supplementary Table 1**. Summary of the 127 targeted deafness genes.

| *Gene* | NS/S | Inheritance | mRNA | Exons |
| --- | --- | --- | --- | --- |
| *ACTG1* | NS | AD | NM_001199954.1 | 6 |
| *ALMS1* | S | AR | NM_015120.4 | 23 |
| *ATP2B2* | NS | AR | NM_001001331.2 | 23 |
| *BSND* | NS/S | AR | NM_057176.2 | 4 |
| *CACNA1D* | NS/S | AR | NM_000720.3 | 49 |
| *CCDC50* | NS | AD | NM_178335.2 | 12 |
| *CDH23* | NS/S | AR | NM_022124.5, | 70 |
| *CEACAM16* | NS | AD | NM_001039213.3 | 7 |
| *CHD7* | S | AD | NM_017780.3 | 38 |
| *CLDN14* | NS | AR | NM_144492.2 | 3 |
| *CLRN1* | NS/S | AR | NM_174878.2 | 3 |
| *COCH* | NS | AD | NM_001135058.1 | 11 |
| *COL11A1* | S | AR/AD | NM_001854.3 | 67 |
| *COL2A1* | S | AD | NM_033150.2 | 53 |
| *COL4A3* | S | AR | NM_000091.4 | 52 |
| *COL4A4* | S | AR | NM_000092.4 | 48 |
| *COL4A5* | S | X-linked | NM_000495.4 | 51 |
| *COL9A1* | S | AR | NM_001851.4 | 38 |
| *COL9A2* | S | AR | NM_001852.3 | 32 |
| *CRYM* | NS | AD | NM_001888.4 | 10 |
| *DFNA5* | NS | AD | NM_004403.2 | 10 |
| *DFNB31* | NS/S | AR | NM_015404.3 | 12 |
| *DFNB59/PJVK* | NS | AR | NM_001042702.3 | 7 |
| *DIABLO* | NS | AD | NM_019887.5 | 7 |
| *DIAPH1* | NS | AD | NM_005219.4 | 28 |
| *DIAPH3* | NS | AD | NM_001258366.1 | 27 |
| *DLX5* | S | AD | NM_005221.5 | 3 |
| *DSPP* | NS | AD | NM_014208.3 | 5 |
| *EDN3* | S | AD | NM_207032.2 | 5 |
| *EDNRB* | S | AD | NM_000115.3 | 8 |

| *ESPN* | NS | AR/AD | NM_031475.2 | 13 |
| --- | --- | --- | --- | --- |
| *ESRRB* | NS | AR | NM_004452.3 | 11 |
| *EYA1* | S | AD | NM_000503.5 | 18 |
| *EYA4* | NS | AD | NM_004100.4 | 20 |
| *FGF3* | S | AR | NM_005247.2 | 3 |
| *FGFR1* | S | AD | NM_023110.2 | 18 |
| *FGFR2* | S | AD | NM_000141.4 | 18 |
| *FGFR3* | S | AD | NM_000142.4 | 18 |
| *FOXI1* | NS/S | AR/AD | NM_012188.4 | 2 |
| *GATA3* | S | AD | NM_001002295.1 | 6 |
| *GIPC3* | NS | AR | NM_133261.2 | 6 |
| *GJA1* | NS | AR | NM_000165.4 | 2 |
| *GJB2* | NS | AR/AD | NM_004004.5 | 2 |
| *GJB3* | NS | AR/AD | NM_024009.2 | 2 |
| *GJB6* | NS | AR/AD | NM_001110219.2 | 5 |
| *GLI3* | S | AD | NM_000168.5 | 15 |
| *GPR98* | NS/S | AR | NM_032119.3 | 90 |
| *GPSM2* | NS | AR | NM_013296.4 | 15 |
| *GRHL2* | NS | AD | NM_024915.3 | 16 |
| *GRXCR1* | NS | AR | NM_001080476.2 | 4 |
| *HGF* | NS | AR | NM_000601.4 | 18 |
| *HOXA1* | S | AR | NM_005522.4 | 2 |
| *HOXA2* | S | AR | NM_006735.3 | 2 |
| *IGF1* | S | AR | NM_001111283.1 | 5 |
| *ILDR1* | NS | AR | NM_001199799.1 | 8 |
| *KCNE1* | NS/S | AR | NM_000219.5 | 4 |
| *KCNJ10* | NS/S | AR | NM_002241.4 | 2 |
| *KCNQ1* | NS/S | AR | NM_000218.2 | 16 |
| *KCNQ4* | NS | AD | NM_004700.3 | 14 |
| *LHFPL5* | NS | AR | NM_182548.3 | 4 |
| *LOXHD1* | NS | AR | NM_144612.6 | 40 |
| *LRP2* | S | AR | NM_004525.2 | 79 |
| *LRTOMT* | NS | AR | NM_145309.5 | 6 |

| *MARVELD2* | NS | AR | NM_001038603.2 | 7 |
| --- | --- | --- | --- | --- |
| *MIR96* | NS | AD | NR_029512.1 |  |
| *MITF* | S | AD | NM_198159.2 | 10 |
| *MSRB3* | NS | AR | NM_198080.3 | 6 |
| *MT-RNR1* | NS | MT | — |  |
| *MT-TE* | S | MT | — |  |
| *MT-TK* | S | MT | — |  |
| *MT-TL1* | S | MT | — |  |
| *MT-TS1* | NS | MT | — |  |
| *MYH14* | NS | AD | NM_001077186.1 | 42 |
| *MYH9* | NS | AD | NM_002473.5 | 41 |
| *MYO15A* | NS | AR | NM_016239.3 | 66 |
| *MYO1A* | NS | AD | NM_001256041.1 | 29 |
| *MYO3A* | NS | AR | NM_017433.4 | 35 |
| *MYO6* | NS | AR/AD | NM_004999.3 | 35 |
| *MYO7A* | NS/S | AR/AD | NM_000260.3 | 49 |
| *NDP* | S | X-linked | NM_000266.3 | 3 |
| *OPA1* | S | AD | NM_015560.2 | 29 |
| *OTOA* | NS | AR | NM_144672.3 | 28 |
| *OTOF* | NS | AR | NM_194248.2 | 47 |
| *OTOG* | NS | AR | NM_001277269.1 | 55 |
| *PAX2* | S | AD | NM_003987.3 | 11 |
| *PAX3* | S | AD | NM_181457.3 | 8 |
| *PCDH15* | NS/S | AR | NM_001142765.1 | 32 |
| *PDSS1* | S | AR | NM_014317.3 | 12 |
| *PDZD7* | NS/S | AR | NM_001195263.1 | 17 |
| *PHEX* | S | X-linked | NM_000444.5 | 22 |
| *POU3F4* | NS | X-linked | NM_000307.4 | 1 |
| *POU4F3* | NS | AD | NM_002700.2 | 2 |
| *PRPS1* | NS | X-linked | NM_002764.3 | 7 |
| *PRRX1* | S | AR | NM_022716.3 | 4 |
| *PTPRQ* | NS | AR | NM_001145026.1 | 45 |
| *RDX* | NS | AR | NM_001260492.1 | 16 |

| *SEMA3E* | S | AR | NM_012431.2 | 17 |
| --- | --- | --- | --- | --- |
| *SERAC1* | S | AR | NM_032861.3 | 17 |
| *SERPINB6* | NS | AR | NM_001297699.1 | 7 |
| *SIX1* | NS/S | AD | NM_005982.3 | 2 |
| *SIX5* | S | AD | NM_175875.4 | 3 |
| *SLC17A8* | NS | AD | NM_139319.2 | 12 |
| *SLC19A2* | S | AR | NM_006996.2 | 6 |
| *SLC26A4* | NS/S | AR | NM_000441.1 | 21 |
| *SLC26A5* | NS | AR | NM_198999.2 | 20 |
| *SLC4A11* | S | AR | NM_001174090.1 | 20 |
| *SMAD4* | S | AD | NM_005359.5 | 12 |
| *SMPX* | NS | X-linked | NM_014332.2 | 5 |
| *SNAI2* | S | AD | NM_003068.4 | 3 |
| *SOBP* | S | AR | NM_018013.3 | 7 |
| *SOX10* | S | AD | NM_006941.3 | 4 |
| *SOX9* | S | AD | NM_000346.3 | 3 |
| *STRC* | NS | AR | NM_153700.2 | 29 |
| *TCOF1* | S | AD | NM_001135244.1 | 26 |
| *TECTA* | NS | AR/AD | NM_005422.2 | 23 |
| *TIMM8A* | NS/S | X-linked | NM_004085.3 | 2 |
| *TJP2* | NS | AD | NM_004817.3 | 23 |
| *TMC1* | NS | X-linked | NM_138691.2 | 24 |
| *TMIE* | NS | AR | NM_147196.2 | 4 |
| *TMPRSS3* | NS | AR | NM_024022.2 | 13 |
| *TNFRSF11B* | S | AD | NM_002546.3 | 5 |
| *TPRN* | NS | AR | NM_001128228.2 | 4 |
| *TRIOBP* | NS | AR | NM_138632.2 | 8 |
| *USH1C* | NS/S | AR | NM_005709.3 | 21 |
| *USH1G* | NS/S | AR | NM_173477.4 | 3 |
| *USH2A* | NS/S | AR | NM_206933.2 | 72 |
| *WFS1* | NS/S | AR/AD | NM_006005.3 | 8 |

NS: Non-Syndromic; S: Syndromic; AR: Autosomal Recessive; AD: Autosomal Dominant; MT: Mitochondrial Inheritance
