# Supplementary material for: A novel MYH14 mutation in a Chinese family with autosomal dominant nonsyndromic hearing loss
Source: BMC Med Genet. 2020 Jul 25;21:154. doi: 10.1186/s12881-020-01086-y (PMC7382048; doi:10.1186/s12881-020-01086-y)
Supplement: Supplementary file 2 — Additional file 2: Table 2. Candidate variants were verified in the family using Sanger sequencing. [file 12881_2020_1086_MOESM2_ESM.docx]

Supplementary Table 2. Candidate variants were verified in the family using Sanger sequencing

| Subject | *MYH14* c.5417C>A | *GJB2* c.109G>A |
| --- | --- | --- |
| Ⅰ-1 | -/- | -/- |
| Ⅰ-2 | M/- | -/- |
| Ⅱ-1 | M/- | -/- |
| Ⅱ-2 | -/- | M/- |
| Ⅱ-3 | -/- | -/- |
| Ⅱ-4 | -/- | -/- |
| Ⅱ-5 | -/- | -/- |
| Ⅱ-6 | -/- | -/- |
| Ⅲ-1 | M/- | M/- |
| Ⅲ-2 | -/- | -/- |
| Ⅲ-3 | -/- | -/- |
| Ⅲ-4 | -/- | -/- |
| Ⅲ-5 | -/- | -/- |
| Ⅳ-1 | M/- | -/- |
| Ⅳ-2 | -/- | -/- |

# *MYH14*, myosin heavy chain 14; *GJB2*, gap junction protein beta 2; M, c.5417C>A; -, wild type.
